# Incidence and prevalence of congenital clubfoot in Apulia: a regional model for future prospective national studies

**DOI:** 10.1186/s13052-023-01559-9

**Published:** 2023-11-14

**Authors:** Raffaella Panza, Federica Albano, Alberto Casto, Cosimo Del Vecchio, Nicola Laforgia, Daniela Dibello

**Affiliations:** 1 Neonatology and Neonatal Intensive Care Unit (NICU), University of Bari Aldo Moro, Bari, Italy; 2 Orthopaedics Unit, Department of Basic Medical Science, Neuroscience and Sensory Organs, School of Medicine, University of Bari Aldo Moro, Bari, Italy; 3 Department of Interdisciplinary Medicine, University of Bari Aldo Moro, Bari, Italy; 4 Unit of Pediatric Orthopaedics and Traumatology, Giovanni XXIII Children’s Hospital, Via Giovanni Amendola, Bari, 70126 Italy

**Keywords:** Clubfoot [MeSH], Clubfeet [MeSH], Congenital talipes equinovarus, Infant, Newborn [MeSH]

## Abstract

**Background:**

Congenital clubfoot is a fairly common and severe congenital malformation, most often of idiopathic origin. A smaller percentage of cases is related to chromosomal abnormalities and genetic syndromes. It is estimated that 0.5/1000 newborns are affected worldwide, with a male to female ratio of 2:1 and greater distribution in developing countries (80%). The “European Surveillance of Congenital Anomalies (EUROCAT)” reported clubfoot prevalence in European newborns, but data regarding Italy are missing or poor. We aim to provide detailed data on clubfoot incidence according to the Apulian Regional Registry on Congenital Malformations and to report current knowledge on clubfoot genetic factors.

**Methods:**

We extrapolated data from the Regional Registry of Congenital Malformations to evaluate incidence and prevalence of congenital clubfoot in Apulia, Italy over a period of four years (2015–2018). We also performed a narrative review focusing on genetic mutations leading to congenital clubfoot.

**Results:**

Over the period from 2015 to 2018 in Apulia, Italy, 124,017 births were recorded and 209 cases of clubfoot were found, accounting for an incidence rate of 1.7/1,000 and a prevalence rate of 1.6/1,000. Six families of genes have been reported to have an etiopathogenetic role on congenital clubfoot.

**Conclusions:**

Incidence and prevalence of congenital clubfoot in Apulia, Italy, are comparable with those reported in the other Italian regions but higher than those reported in previous studies from Europe. Genetic studies to better classify congenital clubfoot in either syndromic or isolated forms are desirable.

**Supplementary Information:**

The online version contains supplementary material available at 10.1186/s13052-023-01559-9.

## Introduction

Congenital clubfoot, also known as talipes equinovarus, is a fairly common and severe congenital malformation, featuring structural defects of the tissues of the foot and lower leg that are responsible for the abnormal positioning of the foot and ankle joints [1]. If left untreated, severe disability and deformities are expected [2]. The etiology of congenital clubfoot is still unknown. To date, the hypothesis of a multifactorial origin is the most widely accepted, since both genetic and environmental factors (i.e. smoke during pregnancy) have an etiopathogenetic role [3].

Eighty percent of cases are idiopathic isolated congenital defects [4], whereas 20% are related to chromosomal abnormalities and genetic syndromes, such as distal arthrogryposis (DA) and myelomeningocele [5].

In about 25% of isolated forms of clubfoot, a positive family history is found, confirming the role of genetic factors [6]. Besides, a higher incidence in monozygotic twins (33%) compared to dizygotic twins (3%) has been described and a 30% risk of heritability of isolated clubfoot has been recently reported [7]. Monochorionic triplets with bilateral isolated clubfoot have also been described [3].

Since the clinical presentation of isolated and syndromic forms may be overlapping, genetic studies in patients with syndromic forms could provide insights on the cause of underlying isolated clubfoot [3].

To describe how often a disease or another health event occurs in a population, different measures of disease frequency can be used. The prevalence reflects the number of existing cases of a disease and can be seen as a measure of disease status: it is the proportion of people in a population having a disease. In contrast to the prevalence, the incidence reflects the number of new cases of disease and can be reported as a risk or an incidence rate. The incidence rate can be calculated by dividing the number of subjects developing a disease by the total time at risk for all people to get the disease. The denominator of this formula includes a measure of time instead of just a number of subjects. The risk is the probability that a subject within a population will develop a given disease, or other health outcome, over a specified follow-up period. It can be calculated by dividing the number of subjects developing the disease over a certain period by the total number of subjects followed over that period.

It is estimated that 0.5/1000 newborns are affected worldwide (150,000–200,000 newborns per year and 7–43 cases of clubfoot/year/million population), with a male to female ratio of 2:1 and greater distribution in developing countries (80%). Kruse and colleagues suggested a reason for this gender difference in the Carter effect [8]. In 50% of cases, it affects both feet; in unilateral clubfoot, the right side is more often involved [9]. According to some epidemiological investigations, major differences in prevalence have been identified between ethnic groups, reaching the highest rates in Maori (7/1000 newborns) [10], Polynesians and Hawaiians (6.8/1000 newborns) [11], and southern Africans (3.5/1000 newborns) [12]. Conversely, the percentages in the Chinese (0.39/1000 newborns) 2, Japanese (0.87/1000 newborns) [13], Asian (0.57/1000 newborns), European (1.2/1000 newborns) [14], and Brazilian population (1.7/1000 newborns) [14] are lower. Recently, the “European Surveillance of Congenital Anomalies (EUROCAT)” reported clubfoot prevalence in European newborns [15], but data regarding Italy are missing or poor, including only the regions of Tuscany and Emilia Romagna [16].

In the latest Italian regional report, Pavone and coll. reported 827 cases of isolated congenital talipes equinovarus (ICTEV) out of 801,324 live births in the Sicilian population from 1991 to 2004, with a prevalence of nearly 1:1000, a male to female ratio of 2:1, and the right foot affected slightly more frequently than the left [17]. In Italy an accurate estimate of regional incidence of congenital clubfoot is possible from The “Certificate of Delivery Care Registry (CeDAP)”, which is a web system providing epidemiological and sociodemographic information about newborns. The current data collection of the CeDAP started on 1 January 2002, following the ministerial Decree no. 349 of 16 July 2001. The certificate is structured into six sections; each section collects specific information regarding birthplace, parents, pregnancy, childbirth, newborns, congenital malformations or the causes of neonatal death. In case of dead-births or fetal malformations, specific information is collected in the certificate. The certificate is completed within the tenth day after birth by the midwife or the doctor in charge.

### Aim of the study

To provide detailed data on clubfoot incidence according to the Apulian Regional Registry on Congenital Malformations and to report current knowledge on clubfoot genetic factors.

## Materials and methods

The Apulian Regional Registry of Congenital Malformations was established on 3rd September 2013 to collect data on congenital malformations, diagnosed either prenatally or by the end of the first year of life. It is regularly updated by healthcare providers across the entire regional territory, hence it provides accurate data on prevalence, incidence and variation across time and space of congenital malformations.

We extrapolated data from the Regional Registry of Congenital Malformations to evaluate incidence and prevalence of congenital clubfoot in Apulia, Italy over a period of four years (2015–2018).

Data were also sorted according to the six Apulian provinces of Brindisi, Taranto, BAT (Barletta-Andria-Trani), Bari, Foggia, and Lecce. Incidence and prevalence were expressed as the new or the total cases of clubfoot per annual births, respectively. Incidence rate was expressed as the number of new subjects diagnosed with clubfoot per year per 1,000 births. Prevalence rate was expressed as the total number of subjects diagnosed with clubfoot per year per 1,000 births.

We also performed a narrative review focusing on genetic mutations leading to congenital clubfoot. An exhaustive search for eligible studies was performed in PubMed, Embase, Medline, Cochrane library and Web of Science databases.

The search string used was “(clubfoot [MeSH term] OR congenital talipes equinovarus[All fields] OR clubfeet [MeSH term]) AND (gene* OR genetic association)”.

Additional studies were sought using references in articles retrieved from searches. Search limits were set for studies published between 1st January 1998 and 29th April 2022 in English language.

## Results

Over the period from 2015 to 2018 in Apulia, Italy, 124,017 births were recorded and 209 cases of clubfoot were found, accounting for an incidence rate of 1.7/1,000 and a prevalence rate of 1.6/1,000. Based on collected data, the highest incidence of congenital clubfoot (1.9/1,000) occurred in 2016 (60 cases/31,681 births) and 2018 (56 cases/29,399 births). The lowest incidence rate (1.4/1,000) was observed in 2014 (43 cases/32,161 births) (Table 1).

**Table 1:** Incidence and prevalence rate (n/1,000 births) of congenital clubfoot in Apulia, from 2015 to 2018

Year	Births	Clubfoot	Incidence	Prevalence
2015	32,161	43	1.4	1.3
2016	31,681	60	1.9	1.8
2017	30,776	50	1.6	1.6
2018	29,399	56	1.9	1.9
**TOTAL**	124,017	209	1.7	1.6

**Table 2:** Incidence rate of congenital clubfoot (n/1,000 births) in Apulia, sorted by the six provinces

Province	2015	2016	2017	2018
FOGGIA	1.2	2.2	1.9	2.6
BAT	0.8	3.0	1.9	2.0
BARI	0.9	1.6	0.7	1.6
BRINDISI	2.3	3.0	1.1	1.9
TARANTO	3.2	1.5	3.2	2.2
LECCE	0.7	1.3	2.2	1.7

**Fig. 1:**
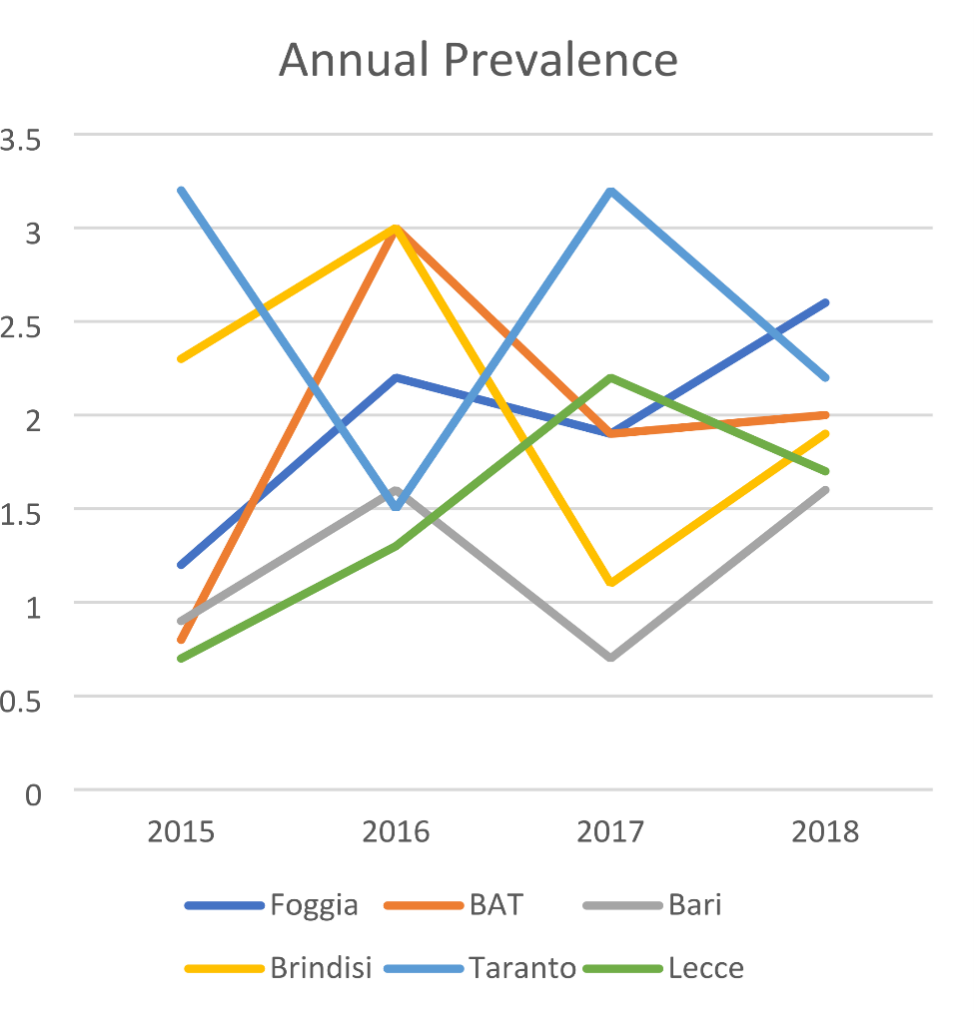
Annual prevalence rate of congenital clubfoot in the six Apulian provinces

Over the studied period, Foggia, BAT (Andria-Barletta-Trani), Bari, and Lecce recorded an increase in the incidence of clubfoot, whereas a decrease was seen in Brindisi and Taranto (Table 2). The trend of the annual prevalence of congenital clubfoot from 2015 to 2018 in the six Apulian provinces is displayed in Fig. 1.

### Narrative review

Six families of genes have been reported to have an etiopathogenetic role on congenital clubfoot.

#### PITX1-TBX4 pathway and Homeobox (HOX) genes

The strongest evidence for the role of genetics regards the PITX1-TBX4 pathway, needed for normal hindlimb development [18, 19]. The PITX1 gene is responsible for rapid changes in pelvic morphology in lower vertebrates and it is involved in foot morphogenesis, being expressed almost exclusively in the hindlimb. In isolated clubfoot phenotypes, dominant segregating mutation in PITX1 [20], inherited microduplications of TBX [4, 21–24] and copy number variants [25] have been described.

In addition to PITX1-TBX4, the involvement of Homeobox (HOX) genes has also been reported. The HOX genes encompass four groups of genes (HOXA-D) controlling limb development throughout the axial and appendicular skeleton [26]. HOXD12 and HOXD13 single nucleotide polymorphisms (SNPs) have been associated with idiopathic clubfoot [27].

Recently, HOXC microdeletions have been shown to overlap with a noncoding region upstream of HOXC13. A missense SNP in HOXC11 in a family with an isolated form of clubfoot and a missense SNP in HOXC12 in clubfoot patients have been reported [28]. The role of insulin-like growth factor binding protein (IGFBP3), HOXD13 gene and genes regulating caspase activity have also been evaluated [3].

#### FSTL gene

FSTL5 is a gene involved in embryonic and postnatal development and it is also a modulator of transforming growth factor beta and bone morphogenetic protein signaling [29].

Studies in mice have shown that FSTL5 is expressed in cartilage cells during later stages of embryonic hind limb development but its abnormalities do not correlate with overt clubfoot-like deformity. This suggests that in the pathogenesis of idiopathic talipes equinovarus, FSTL5 mutations affect other cell lines such as neural cells.

#### SHOX gene duplication

The SHOX gene is part of a large family of homeobox genes, which act during early embryonic development to control the formation of body structures being essential for the growth and maturation of long bones.

One copy of the SHOX gene is located on each sex chromosome in the pseudo-autosomal region. Microduplications of the pseudo-autosomal chromosome region Xp22.33 (Par1) containing SHOX have been found in about 1% of clubfoot patients [30].

#### PMA mice

The PMA (peroneal muscular atrophy) mouse is a good animal model for disorders like arthrogryposis multiplex congenita or congenital clubfoot deformity.

In PMA mice, the peroneal branches of the sciatic nerves are absent. The defect is related to reduced growth of sciatic nerve lateral motor column (LMC) neurons and to an upregulation of LIM-domain kinase 1 (Limk1).

Genetic analyses showed that the mutation acts in the EphA4–Limk1–Cfl1/cofilin–actin pathway [31].

#### Muscle contractile genes

Alterations in muscle fibers and a growth disorder with a disproportionate amount of type I fibers in the posterior and medial muscle groups were found, suggesting the presence of an abnormality in neural development. Involvement of the tendon sheaths of the finger flexors and posterior tibial tendons can also be found, which shows signs of cellular hypoplasia with smaller cells and less cytoplasmic volume. [9]

Newborns with idiopathic talipes equinovarus show calf muscle hypoplasia at birth, suggesting the involvement of genes related to muscle development. Accordingly, alterations of genes encoding for muscle proteins (MYH3, TPM2, TNNT3, TNNI2, and MYH8) cause congenital contractures. Other genes involved are those encoding myosin heavy chains 3 and 8 (MYH3, MYH8), troponin I and T (TNNI2, TNNT3), and tropomyosin (TPM2). [32]

#### FLNB and ECM proteins

Filamin B (FLNB) is a protein that binds actin in a dynamic structure. FLNB missense mutations have been associated with isolated clubfoot.

Genetic analyses revealed mutations in genes involved in various cellular processes, including proliferation, apoptosis, differentiation, and extracellular matrix formation and remodeling. Genes in the collagen family have also been linked to idiopathic congenital talipes equinovarus. Mutations in genes encoding the ECM proteins COL9A1, COL9A2, COL9A3, COMP and MATN3, as well as the transmembrane glycoprotein involved in matrix organization, SLC26A2, have been associated with clubfoot. Mutations in peroxisomal biogenesis (PEX) factors, including PEX26, are also included in the pathogenesis of clubfoot [33].

## Discussion

The present study is based on a retrospective study focusing on the Italian pediatric population affected by clubfoot of a single Italian region (Apulia), where a Regional registry has been instituted in 2013, since to date national data from a national registry are still lacking [34].

According to CeDAP certificates, the incidence rate of congenital clubfoot varies significantly across Italian regions, ranging from 2.8/1,000 (Liguria) to 23.4/1,000 (Valle d’Aosta) [34].

We report a prevalence rate of 1.6/1,000 and an incidence rate of 1.7/1,000 for congenital clubfoot over the period from 2015 to 2018 in Apulia.

A similar prevalence rate was reported from the registries of Umbria and Abruzzo, while the lowest prevalence rate are reported from Campania (0.1/1,000) and Sardinia (0.4/1,000).

Only few studies on the prevalence of clubfoot have been carried out worldwide.

A European study was conducted using data from the EUROCAT network from 1995 to 2011. The total prevalence of congenital clubfoot was 1.13/1,000 births (95% CI 1.10–1.16). The prevalence of congenital clubfoot without chromosomal abnormalities was 1.08/1,000 births (95% CI 1.05–1.11), and the prevalence of isolated congenital clubfoot was 0.92/1,000 births (95% CI 0.90–0.95). However, significant geographical differences in prevalence emerge in this study: from 0.44/1,000 births in Tuscany and 0.45/1,000 births in the Basque Country to 1.68/1,000 births in Wales. A decreasing trend over time was observed for both incidence and prevalence. The majority of cases were isolated congenital clubfoot (82%), whereas 11% had associated major congenital anomalies. The prenatal detection of isolated congenital clubfoot rate increased over time, reaching a peak of 22% [35].

The overall prevalence of congenital clubfoot in EUROCAT study was comparable with those observed in other studies: 1.8/1,000 livebirths for all congenital clubfoot cases and 1.1/1,000 births for isolated congenital clubfoot in a study in Southern Australia [36] and 1.14/1,000 livebirths for isolated congenital clubfoot in Iowa [35, 37].

In a different study based on The National Swedish register, Sweden reported 828 isolated clubfeet and 77 non-isolated clubfeet from 2016 to 2019. The prevalence rate was 1.24/1,000 live births for isolated clubfoot, and 0.11/1,000 for non-isolated cases. In total 612 children with isolated or non-isolated clubfoot were recorded, accounting for a prevalence rate of 1.35/1,000. [38]

From 2012 to 2015 a retrospective descriptive study based on the Sri Lanka Clubfoot Program database reported 87% idiopathic clubfoot, 48% bilateral deformities and 13% non-isolated clubfoot. [39]

An accurate estimate of incidence and prevalence is essential not only for epidemiological-statistical purposes but also to improve genetic diagnostic panels. Unfortunately, our data lack the distinction in isolated or syndromic forms. Moreover, available literature on the etiology of clubfoot has important limitations due to considerable heterogeneity. To date, there is no consensus on which genetic abnormality should be regarded as the main causative target. Besides the PITX1-TBX4 pathway and Homeobox (HOX) genes, whose role is historically recognized, neural cell line mutations (e.g. FSTL gene), SHOX gene and abnormalities in cellular matrix cells, muscle fibers proteins and peroxisomes deserve some importance. However, further international collaborative studies are strongly encouraged to gain a deeper knowledge on genetic causative factors and provide genetic diagnostic panels.

### Strenghts and limitations

The present study provides accurate and reliable data from an Italian region and poses the basis for a larger work aimed to estimate the national extent of this pathology. Limitations are given by the retrospective nature of the study, the narrow time frame considered (2015–2018), and the lack of distinction in isolated or syndromic forms of congenital clubfoot.

## Conclusions

Congenital clubfoot is an emerging malformation whose incidence and prevalence in Apulia, Italy, range from 1.4 to 1.9 and 1.3 to 1.9 per 1,000 births, respectively. Such values are comparable with Italian data from the CeDAP registry but higher than those reported in previous studies from Europe. An accurate prospective national study is warranted to improve the knowledge on incidence and prevalence on a larger scale. Genetic studies to better classify congenital clubfoot in either syndromic or isolated forms are also desirable.

### Electronic supplementary material

Below is the link to the electronic supplementary material.

Supplementary Material 1

## Data Availability

All relevant data are included in the article.

## Abbreviations

CeDAP: Certificate of Delivery Care Registry
DA: Distal arthrogryposis
EUROCAT: European Surveillance of Congenital Anomalies
FLNB: Filamin B
HOX: Homeobox
ICTEV: Isolated congenital talipes equinovarus
LMC: Lateral motor column
PEX: Peroxisomal
PMA: Peroneal muscular atrophy
