# Supplementary material for: Incidence and prevalence of congenital clubfoot in Apulia: a regional model for future prospective national studies
Source: Ital J Pediatr. 2023 Nov 14;49:151. doi: 10.1186/s13052-023-01559-9 (PMC10648723; doi:10.1186/s13052-023-01559-9)
Supplement: Supplementary file 1 — Supplementary Material 1 [file 13052_2023_1559_MOESM1_ESM.docx]

Dear Editor,

we are pleased to submit to your attention the paper titled: **“Incidence and prevalence of congenital clubfoot in Apulia: a regional model for future prospective national studies”.**

This paper aims at providing detailed data on clubfoot incidence according to the Apulian Regional Registry on Congenital Malformations and reporting current knowledge on clubfoot genetic factors.

We extrapolated data from the Regional Registry of Congenital Malformations to evaluate incidence and prevalence of congenital clubfoot in Apulia, Italy over a period of four years (2015-2018). We also performed a narrative review focusing on genetic mutations leading to congenital clubfoot.

Over the period from 2015 to 2018 in Apulia, Italy, 124,017 births were recorded and 209 cases of clubfoot were found, accounting for an incidence rate of 1.7/1,000 and a prevalence rate of 1.6/1,000. Six families of genes have been reported having a etiopathogenetic role of congenital clubfoot.

Incidence and prevalence of congenital clubfoot in Apulia, Italy, are comparable with those reported in the other Italian regions but higher than those reported in previous studies from Europe. Genetic studies to better classify congenital clubfoot in either syndromic or isolated forms are desirable.

There are no prior publications or submissions with any overlapping information, including studies and patients.

The authors declare no conflict of interest, real or perceived.

All the authors have written the work and each author has seen and approved the submission of this version of the manuscript.

Best Regards,

Raffaella Panza
